# P-1938. The Size of Pulmonary Invasive Mold Infection and Underlying Host Diseases

**DOI:** 10.1093/ofid/ofaf695.2106

**Published:** 2026-01-11

**Authors:** Hyeon Mu Jang

**Affiliations:** Asan medical center, Seoul, Seoul-t'ukpyolsi, Republic of Korea

## Abstract

**Background:**

Computed tomography (CT) is important to diagnose pulmonary invasive mold infection (PIMI). The radiologic criteria by the 2020 European Organization for Research and Treatment of Cancer (EORTC) include dense, well-circumscribed lesion, air crescent sign, cavity, and consolidation. The dense, well-circumscribed lesion(s) are classified into nodules or masses by the cut-off of 3cm, although there is no such cutoff for the other types of lung lesions. Moreover, there are limited data on the size of PIMI and its clinical characteristics. We thus evaluated the size of the representative lesion, dividing them into two groups (≤3cm, >3cm) and its clinical characteristics including underlying host diseases.

**Table 1.**
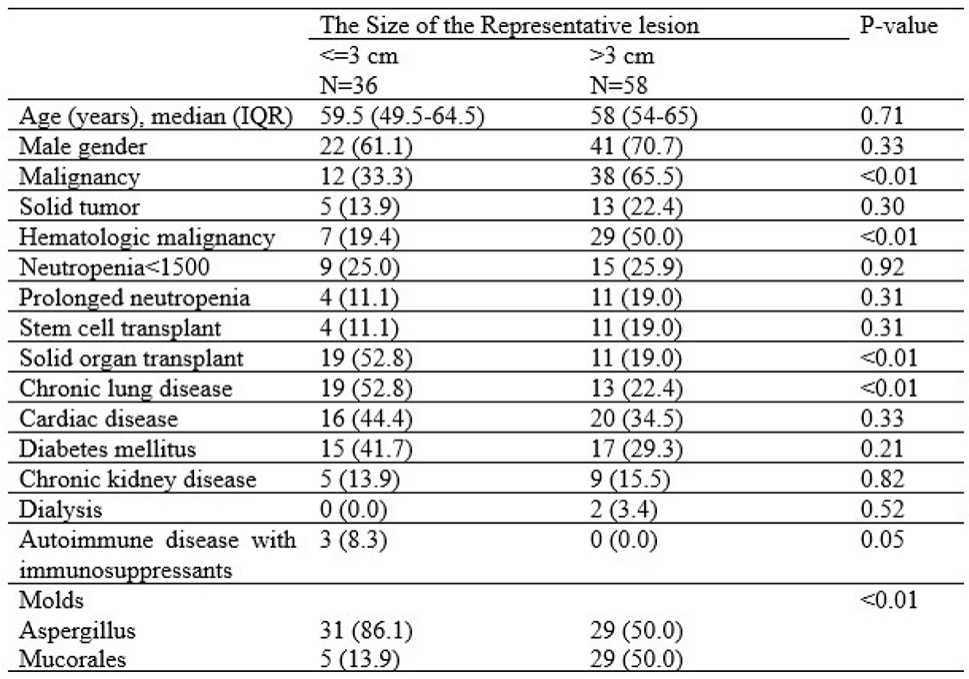
Clinical characteristics of invasive mold infection by the size (≤3cm, >3cm)

**Figure 1.**
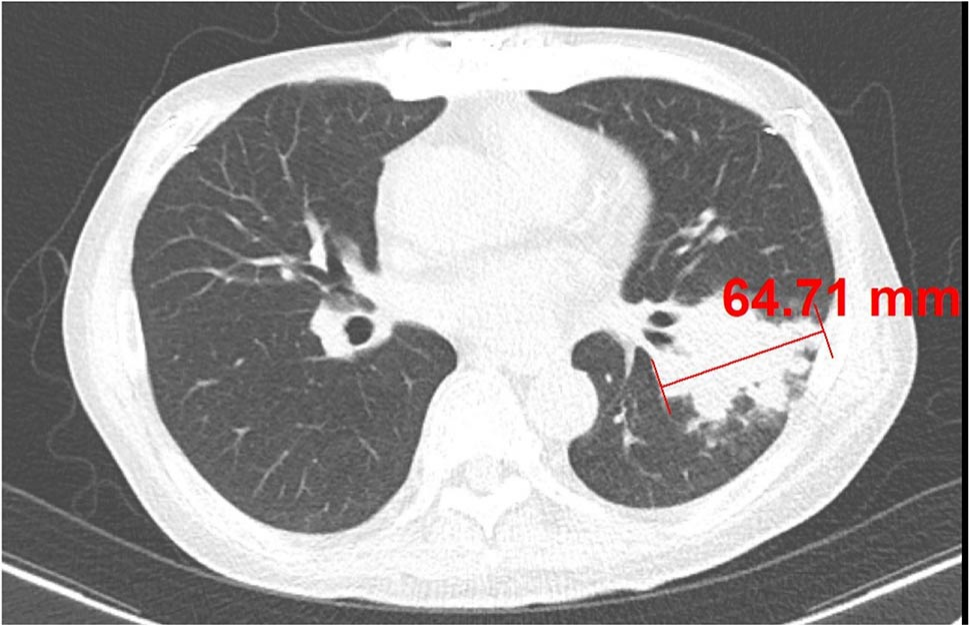
The size of representative lesion It was defined as the long diameter of the mass or consolidation (excluding GGO)

**Methods:**

We reviewed all adult patients, at a 2700-bed tertiary hospital, in Seoul, South Korea, from January 2003 to June 2024, who were diagnosed with proven invasive pulmonary aspergillosis (IPA) or proven pulmonary mucormycosis (PM) by EORTC criteria. The representative lesion was defined as a lesion with the largest distribution and representative of the entire lesion. The size of the representative lesion was defined as the long diameter of the mass or consolidation (excluding GGO).

**Results:**

A total of 94 patients with proven PIMIs (60 IPA and 34 PM) were analyzed. Of the 94 patients, 36 patients (38.3%) had PIMIs ≤3cm and 58 patients (61.7%) had lesions >3cm. The bigger lesions ( >3cm) were significantly more associated with hematologic malignancies (19% [7/36] vs 50% [29/58]; 0< 0.01) whereas the smaller lesions (≤3cm) were more associated with solid organ transplant (53% [19/36] vs 19% [11/58]; p< 0.01) and chronic lung diseases (53% [19/36] vs 22% [13/58]; p < 0.01). The smaller lesions (≤3cm) were significantly more associated with IPA (86% [31/36] vs 50% [29/58]; p < 0.01).

**Conclusion:**

More than 60% of proven PIMI were bigger than 3cm. The bigger lesions ( >3cm) were significantly more associated with hematologic malignancies and PM whereas the smaller lesions were associated with solid organ transplant, chronic lung diseases and IPA.

**Disclosures:**

All Authors: No reported disclosures

